# Terpenes and terpenoids as main bioactive compounds of essential oils, their roles in human health and potential application as natural food preservatives

**DOI:** 10.1016/j.fochx.2022.100217

**Published:** 2022-01-19

**Authors:** Ayu Masyita, Reka Mustika Sari, Ayun Dwi Astuti, Budiman Yasir, Nur Rahma Rumata, Talha Bin Emran, Firzan Nainu, Jesus Simal-Gandara

**Affiliations:** a Faculty of Pharmacy, Hasanuddin University, Makassar 90245, Sulawesi Selatan, Indonesia; b Department of Chemistry, Faculty of Mathematics and Natural Sciences, Universitas Sumatera Utara, Medan 20222, Sumatera Utara, Indonesia; c Cellulosic and Functional Materials Research Centre, Universitas Sumatera Utara, Jl. Bioteknologi No.1, Medan 20155, Indonesia; d Sekolah Tinggi Ilmu Farmasi Makassar, Makassar 90242, Sulawesi Selatan, Indonesia; e Department of Pharmacy, BGC Trust University Bangladesh, Chittagong 4381, Bangladesh; f Universidade de Vigo, Nutrition and Bromatology Group, Department of Analytical Chemistry and Food Science, Faculty of Science, E32004 Ourense, Spain

**Keywords:** Essential oil, Anticancer, Antimicrobial, Anti-inflammatory, Food preservatives, Terpenes, Carvacrol (PubChem CID: 10364), Linalool (PubChem CID: 6549), Hinokitiol (PubChem CID: 3611), Bakuchiol (PubChem CID: 5468522), Limonene (PubChem CID: 22311), Eugenol (PubChem CID: 3314), Myrcene (PubChem CID: 31253), α-Terpineol (PubChem CID: 17100), Atractylone (PubChem CID: 3080635), Carvone (PubChem CID: 7439)

## Abstract

Essential oils (EOs) are volatile and concentrated liquids extracted from different parts of plants. Bioactive compounds found in EOs, especially terpenes and terpenoids possess a wide range of biological activities including anticancer, antimicrobial, anti-inflammatory, antioxidant, and antiallergic. Available literature confirms that EOs exhibit antimicrobial and food preservative properties that are considered as a real potential application in food industry. Hence, the purpose of this review is to present an overview of current knowledge of EOs for application in pharmaceutical and medical industries as well as their potential as food preservatives in food industry.

## Highlights

• Terpenes and terpenoids are the main bioactive compounds of essential oils (EOs).
• EOs and their major constituents confer several biological activities.
• EOs are potential as natural food preservatives.

## Introduction

The notion to promote the use of natural products in daily life have spread worldwide in the last several decades. Of all, essential oils (EOs) have been one of the most utilized natural products (Carpena, Nuñez-Estevez, Soria-Lopez, Garcia-Oliveira, & Prieto, 2021). Essential oils are highly concentrated hydrophobic liquid derived from a variety of plants and defined based on their chemical and physical properties. The pharmacological effect of EOs have been extensively examined: ranging from antimicrobial (Burt, 2004, Falleh et al., 2020), antihelminthic (Inouye, Takizawa, & Yamaguchi, 2001), antiviral (Silva, Figueiredo, Byler, & Setzer, 2020), antiulcer (Dordević et al., 2007), antioxidant (Falleh et al., 2020, Mimica-Dukić et al., 2003), anti-inflammatory (Silva et al., 2015), insecticide (Isman & Machial, 2006), larvacidal (Jantan, Ping, Visuvalingam, & Ahmad, 2003), immunomodulatory (Mediratta, Sharma, & Singh, 2002), and antinociceptive properties (Abdollahi, Karimpour, & Monsef-Esfehani, 2003).

Essential oils have long been used as flavorings in the food industry (Pandey, Kumar, Singh, Tripathi, & Bajpai, 2017). Of thousands different EOs known at present, around 300 are commercially marketed in the flavor and fragrances products (Hyldgaard, Mygind, & Meyer, 2012). In addition to their aromatic qualities, the antimicrobial properties of EOs against a wide range of microorganisms have also provided convincing evidence that EOs are suitable candidates to be used as natural food preservatives (Falleh et al., 2020). Among all chemical components of EOs, terpenes and terpenoids have been comprehensively studied and reported to play key roles in humans health (Perveen, 2018).

Terpenes (pinene, myrcene, limonene, terpinene, *p*-cymene) are characterized as compounds with simple hydrocarbons structures while terpenoids (oxygen-containing hydrocarbons) are defined as modified class of terpenes with different functional groups and oxidized methyl groups moved or removed at various positions (Perveen, 2018). Terpenes have been reported to exert antimicrobial activities against both the antibiotic-susceptible and antibiotic-resistant bacteria, mainly via their abilities to promote cell rupture and inhibition of protein and DNA synthesis (Álvarez-Martínez, Barrajón-Catalán, Herranz-López, & Micol, 2021). Carvacrol, carvone, eugenol, geraniol, and thymol were among the terpenes that demonstrated antibacterial action against *Staphylococcus aureus* (Gallucci et al., 2009). In addition, terpenoids have been shown as one of secondary metabolites produced by aromatic and medicinal plants that played a key role in disease resistance. For example, monoterpenoids are antibacterial in nature, causing disruption in microbe multiplication and development, as well as interfering with their physiological and metabolic activities (Burt, 2004). Some botanical compounds, such as azadirachtin, carvone, menthol, ascaridol, methyl eugenol, toosendanin, and volkensin, have been shown to yield antimicrobial and antifungal properties, as well as insect pest repellent properties (Isman and Machial, 2006, Pandey et al., 2016, Pandey et al., 2017).

**Fig. 1:**
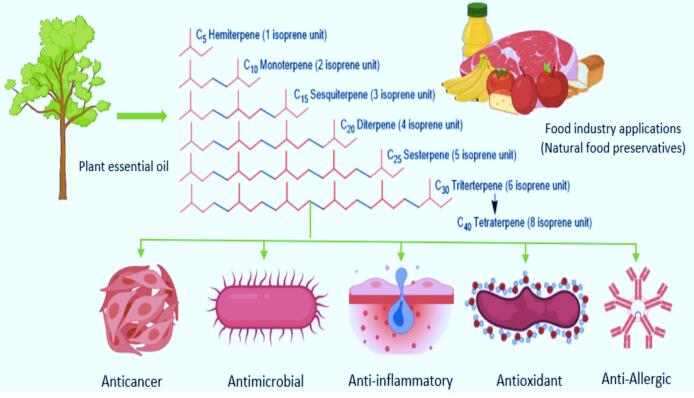
The potential benefits of terpenes and terpenoids found in EOs.

In addition to their various pharmacological effects in humans, the antimicrobial capabilities of EOs’ terpenes and terpenoids against foodborne microbes and their beneficial use in food as flavoring additives shall serve as excellent alternatives to the standard bactericides and fungicides currently used in the food industry (Perricone, Arace, Corbo, Sinigaglia, & Bevilacqua, 2015). This review will thus provide an overview to the current knowledge about the potential role of terpenes and terpenoids, as main bioactive compound of EOs, in human health and their industrial potential as natural food preservatives (Fig. 1). This, in turn, shall provide prompt opportunities to cultivate more and better ideas and research avenues that can promote extensive pharmaceutical applications of EOs’ constituents in the daily life.

## Bioactive compounds of essential oils

Essential oils are concentrated liquids of complex mixtures of aromatic hydrophobic oily volatile compounds and can be extracted from different parts of plants such as bark, buds, flowers, fruits, leaves, peels, roots, seeds, twigs, or whole plant from a single botanical source (Falleh et al., 2020, Stephane and Jules, 2020). International Organization for Standardization (ISO) defines that EOs are products obtained from raw material of vegetables by pressing or distillation processes (Stevanovic, Sieniawska, Glowniak, Obradovic, & Pajic-Lijakovic, 2020).

Essential oils possess a strong odor and usually colorless especially when fresh, but some exceptions are known such as pale yellow (yellow mandarin), blue (chamomile), orange (sweet orange), and green (bergamot). EOs may be easily oxidizable with age by air, heat, or light exposure which proceed to the dark color. Hence, EOs need to be stored in a cool and dry place (Stephane & Jules, 2020). Structurally, the chemical constituents of EOs can be classified into four groups: terpenes, terpenoids, phenylpropanoids, and other constituents (Hyldgaard et al., 2012, Pandey et al., 2017).

### Terpenes

**Fig. 2:**
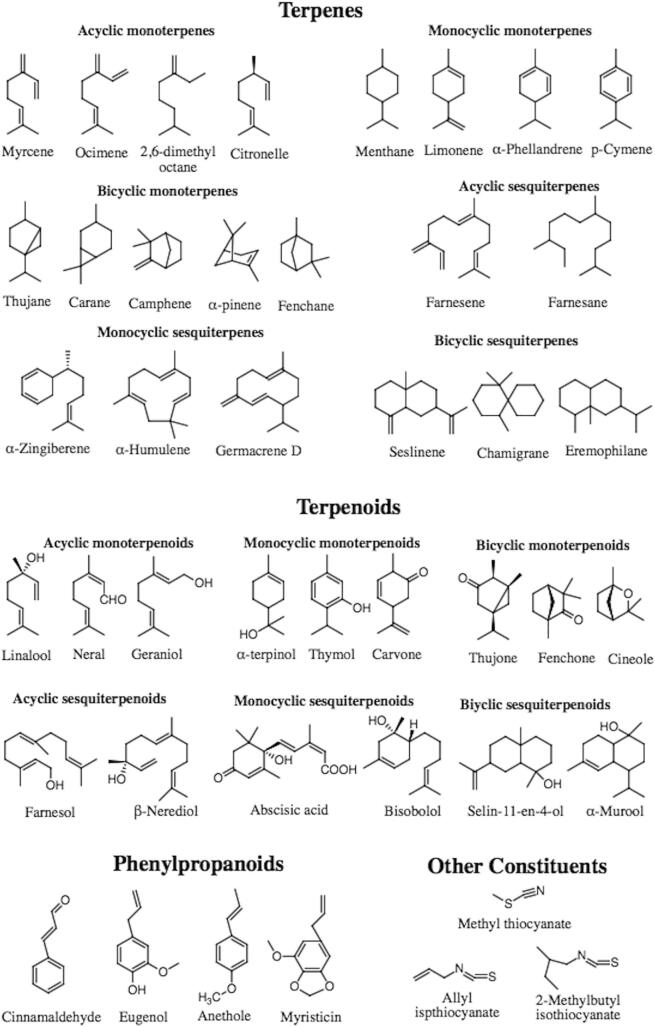
The chemical structures of bioactive compounds of EOs.

Terpenes or isoprenoids are the major constituents found in EOs with molecular structures containing carbon backbones of 2-methylbuta-1,3-diene (isoprene units) which can be rearranged into cyclic structures (Hyldgaard et al., 2012). The number of isoprene units are primarily responsible for structural diversity of terpenes. Hemiterpenes are formed by one isoprene unit (C5), monoterpenes (C10), sesquiterpenes (C15), diterpenes (C20), triterpenes (C30), and tetraterpenes (C40) (Bhavaniramya, Vishnupriya, Al-Aboody, Vijayakumar, & Baskaran, 2019). Hemiterpenes are a minor part of terpenes found in EOs. The most outstanding HT, isoprene which is emitted from the herbs and leaves of many trees such as conifers, oaks, poplars, and willows. Examples of hemiterpenes include angelic, tiglic, isovaleric, and senecioic acids. Monoterpenes are the predominant components of EOs (90%), followed by sesquiterpenes (Falleh et al., 2020). Diterpenes, triterpenes, and tetraterpenes with their oxygenated derivatives are also detected in small amounts (Stephane & Jules, 2020). Examples of bioactive compounds of EO are presented in Fig. 2.

### Terpenoids

Terpenoids are another type of terpenes containing oxygen molecules that are constructed via biochemical modifications (removal or addition of methyl groups) (Pandey et al., 2017). Terpenoids can be divided into alcohols, aldehydes, esters, ether, epoxides, ketones, and phenols. Examples of terpenoids are: carvacrol, citronellal, geraniol, linalool, linalyl acetate, piperitone, menthol, and thymol (Hyldgaard et al., 2012). These bioactive compounds confer several biological activities such as anticancer (Potočnjak, Gobin, & Domitrović, 2018), anti-allergic (Kobayashi et al., 2016), antibacterial (Guimarães et al., 2019), and antioxidant (Wang, Chen, & Hou, 2019).

Terpenes and terpenoids are synthesized by the mevalonic acid (MVA) pathway in the cytosol and the 2C-methyl-d-erythritol-4-phosphate (MEP) pathway in the plastid for the formation of precursors: isopentenyl pyrophosphate (IPP) and dimethylallyl pyrophosphate (DMAPP) (Oldfield and Lin, 2012, Stephane and Jules, 2020).

### Phenylpropanoids

Phenylpropanoids are synthesized by the shikimic acid pathway and their basic structure from the six-carbon aromatic phenol group linked usually to the three-carbon propene tail of cinnamic acid, oxygenated in the third/fourth/fifth position frequently possess a carbon–carbon double bond (Stevanovic et al., 2020). Examples of phenylpropanoids such as anethole, cinnamaldehyde, eugenol, isoeugenol, myristicin, safrole, and vanillin. In a recent study, anethole has been reported to yield anticancer activity (Contant, Rouabhia, Loubaki, Chandad, & Semlali, 2021). Moreover, myristicin has been described to exert antiproliferative and anti-inflammatory properties (Seneme, Dos Santos, Silva, Franco, & Longato, 2021) and safrole has been reported to promote diverse biological activities, such as antidiabetic, antimicrobial, analgesic, and antifungal activities (Eid & Hawash, 2021).

### Other constituents

EOs contain several derivatives of amino acids such as alanine, isoleucine, leucine, valine, and methionine. Polyketides, lipids, and sulfur derivatives are rarely found in EOs such as jasmonic acid, methyl jasmonate, cis-jasmone, (Z)-3-hexenal, allicin (Pandey et al., 2017, Stevanovic et al., 2020).

## Green extraction and purification methods of terpenes and terpenoids

Extraction is the most essential stage in extracting and purifying active compounds from natural sources. Natural chemicals can be extracted via maceration, infusion, soxhletation, percolation, or digesting methods (Giacometti et al., 2018). Due to growing energy prices, CO_2_ emissions, and other associated environmental issues, extraction techniques for the chemical, food, and pharmaceutical sectors have received much interest in recent years. One of the most popular topics in this approach is the development of methods and procedures to achieve maximal extraction at a low cost and in an environmentally friendly. In general, the methods involve the following steps (Fig. S2, Supplementary material): (a) breaking plant cells to release their chemical constituents; (b) extracting the sample using a suitable solvent—or through distillation or compound trapping; (c) separating the desired terpene from undesired contents of extracts that confound analysis and quantification; and (d) analyzing the product using an appropriate method (Asl & Khajenoori, 2021).

In this chapter, the processes in isolation of terpene and terpenoid chemicals in plants and the technique of determining structure elucidation were presented (Fig. S3, Supplementary material). The pre-treatment stage is the most time-consuming. This stage is determined by the physical condition of the sample, which might be solid or liquid. Several techniques are employed to decrease the size of the solid sample, including steaming, dry distillation, and heating, which is subsequently milled. For liquid samples, another option is to employ solvent treatment or chemical treatment. This method is critical in sample preparation to improve extraction efficiency. Traditional terpene and terpenoids extraction methods include maceration, soxhlet extraction, solvent extraction, pressurized liquid extraction, and hydrodistillation (Isidore, Karim, & Ioannou, 2021). Solvent extraction used such as petroleum ether, ether, and hexane for isolating mono and sesquiterpenoids. Less polar sesquiterpene lactones, diterpenes, sterols, and triterpenoids may also be extracted using ether and chloroform. Oxygenated diterpenoids, sterols, and triterpenoids are found in the ethyl acetate and acetone extracts. Highly oxygenated, polar triterpenes, as well as triterpenoid, are extracted using ethanol, methanol, and water. Total extraction using polar solvents including acetone, aqueous methanol (80%), and ethanol, followed by re-extraction with hexane, chloroform, and ethyl acetate, resulted in sequential extraction of terpenoids. Whereas, modern methods include supercritical carbon dioxide extraction, static headspace (HS) extraction, microwave-assisted hydrodistillation (MWHD), supercritical fluid extraction (SFE) and ultrasound-assisted extraction (UAE). Supercritical fluid extraction (SFE) with carbon dioxide as the solvent is considered a “green extraction” method in comparison to traditional extraction methods because it uses a solvent that is essentially non-toxic, has a low potential for artifact formation, and CO_2_ can be obtained in high purity suitable for the production of food grade extracts. The inclusion of polarity modifiers such as EtOH, as well as the development of SFE apparatus capable of producing pressures in excess of 600 bar, enabled the extraction of several intermediate polarity compounds (Essien, Young, & Baroutian, 2020). UAE was useful for the green production of solvent-free terpenoid-rich peppermint extracts. MWHD is better than traditional techniques since it requires fewer extraction times and less solvent. It operates at atmospheric pressure, utilizing microwaves and gravity without solvents (Pavlić et al., 2021). Furthermore, recent report suggested that HS was the most efficient extraction method to recover terpenoids from various food matrices, compared to hydrodistillation and pressurized liquid extraction (Triaux, Petitjean, Marchioni, Steyer, & Marcic, 2021). Compound extraction generates a complicated mixture of compounds. Specific compounds are now isolated using purification methods. Purification procedures varies due to variations in terpene and terpenoids structure, and they are depending on the chemical characteristics of the target molecule, the physical quality and amount of initial plant material, and the availability of equipment and reagents. The properties of a particular terpene will define the optimum extraction method (Yang et al., 2020, Zhang and Hong, 2020). This is followed by compound identification, characterization, and authentication namely Thin Layer CT (Chromatostrip), High-Performance Counter-Current Chromatography (HPCCC), Liquid-Liquid Extraction (LLE), High Performance Liquid Chromatography UV (HPLC-UV), Gas Chromatography Mass Spectrometry (GC–MS), Liquid Chromatograph (LC), High Performance Thin Layer Chromatography (HPTLC), Fast-GC Analysis and Ultra-High-Performance Liquid Chromatography (UHPLC) (Rodríguez-Llorente et al., 2020, Uwineza and Waśkiewicz, 2020).

## Role of terpenes and terpenoids on human health

In recent decades, numerous studies have reported that terpenes and terpenoids are essential in supporting human health. These bioactive compounds consisting of several isoprene units is the largest class of organic compounds produced in the EOs of various plants. It has a significant role to treat various types of diseases, in many studies in vitro and in vivo using as anticancer agents, antimicrobial, anti-inflammatory, antioxidants, antiallergic, neuroprotective, anti-aggregator, anti-coagulation, sedative and analgesic through the activity of monoterpenes, sesquiterpenes, diterpenes, triterpenes, and tetraterpenes and glycoside compounds (Zhao, Jiang, Li, Yan, & Zhang, 2016). The content of this compound can be found in several nutritional and health products of humans because it is a source of vitamins A, E, K, and coenzyme Q10. Even carotenoid and tocopherol compounds in this group are essential sources of vitamins, mostly in animals, including humans (Rodriguez-Concepcion et al., 2018). The application of terpenes compounds in everyday human life and health is widely used in pharmaceutical, nutraceutical, food and beverage products, cosmetics, perfumes, synthetic chemicals, aroma and flavor additives, rubber products, and the biofuel industry (Tetali, 2019).

### Anticancer

Cancer is a regenerative disease that affects anybody part's abnormal and uncontrolled cell growth. It can attack or spread to various other parts of the body and interfere with the normal function of homeostasis of its host cells (Sung et al., 2021). Today worldwide, it is one of the leading causes of morbidity and mortality in the contemporary world, number second after cardiovascular disease and being one in six human deaths and constitutes 30% of all premature deaths occurring in adults (30–69 years). In addition, the number of cancer deaths is predicted to continue to grow and multiply. This cancer is caused by the influence of internal factors, inability, and external factors in lifestyle patterns carried out every day. A food diet can protect from risk factors for carcinogenesis in the human body (El-Sherif, El-Sherif, Taylor, & Ayakannu, 2021).

Terpenes and terpenoids are the largest class of organic compounds produced in the EOs of various plants and have a significant role in the prevention of malignancies in cancer (Silva, Nascimento, Silva, Silva, & Aguiar, 2021). The anticancer potencies of terpenes and terpenoids have been reported extensively in recent years (Table 1). Sheikh, Sarker, Kamarudin, and Mohan (2017) studied the effect of citral on the proliferation of human colorectal cancer HCT116 and HT29 cells. They found that citral decreased the expression of Bc-2 and Bcl-xL while inducing p53 protein phosphorylation and Bax expression. Citral also elevated mitochondrial-mediated apoptosis via augmentation of intracellular ROS and attenuated the total GSH levels (Sheikh et al., 2017).

**Table 1:** Examples of terpene compounds in essential oils with potential anticancer effects.

Classes	Compounds	Plant	Target cancers	Cancer effects	Type of assay	Reference
Monoterpene hydrocarbons	Myrcene	PC	Human lung adenocarcinoma (A549)	Increased apoptosis via caspase induction (IC_50_ 0.5 μg/ml)	MTT	(Bai & Tang, 2020)
Cyclic monoterpene	Limonene	Navel orange (Essential oil)	Lung adenocarcinoma (A549)	Reduced cell proliferation (IC_50_ 22.10 µg/ml)	MTT	(Yang et al., 2017)
Bicyclic monoterpene	α-Pinene	PC	Human liver cancer (HepG2)	Reduced cell growth (IR 39.3%)	MTT	(Xu et al., 2018)
Acyclic monoterpene	Linalool	PC	Human ovarian cancer (SKOV3ip1, A2780, HeyA8)	Increased apoptotis and cytotoxicity	MTT	(Han, Cho et al., 2016)
Oxygenated monoterpene	α-Thujone	*Thuja occidentalis* L.	Human glioblastoma multiforme (T98G), and human glioblastoma (U-87 MG)	Induction of cell death, reduced proliferation and invasive	TB exclusion	(Pudełek et al., 2019)
Terpineols Monoterpene	α-Terpineol	Navel orange (Essential oil)	Lung adenocarcinoma (A549)	Increased caspase-dependent cell death and reduced proliferation (IC_50_ 51.37 µg/ml for 24 h)	MTT	(Yang et al., 2017)
Oxygenated monoterpene	1,8-cineole	PC	Head and neck squamous cell carcinoma (HNSCC)	Reduced proliferation, Wnt/β-catenin activity	MTT	(Roettger et al., 2017)
Cyclic monoterpene	α-Phellandrene	PC	Melanoma(B-16/F-10), and Murine (S-180)	Antinociceptive and tumor-reducing effect (CI_50_ 436.0 and 217.9 μg/ml)	MTT	(Pinheiro-Neto et al., 2021)
Bicyclic Monoterpene	3-carene	Gannan Navel (Essential oil)	Human lung cancer (A549)	Reduced proliferation (IC_50_ 70.80 µg/ml)	MTT	(Yang et al., 2017)
Hydroxylated monoterpene	Perillyl alcohol	PC	Hepatoma cell	Suppress cell invasion and migration	Transwell	(Ma et al., 2016)
Terpenophenol	Bakuchiol	PC	Human gastric cancer (NUGC3)	Increased cell death and reduced cancer cell viability (IC_50_ 120 µg/ml for 24 h)	MTT	(Lv & Liu, 2017)
Monoterpenoid phenol	Carvacrol	PC	Human Caucasian gastric adenocarcinoma (AGS)	Increased cell apoptosis, inhibited proliferation, GSH-reducing effects on cell (IC_50_ 82.57 μmol/l)	CellTiter-Glo	(Günes-Bayir et al., 2017)
Citral isomers	Geranial, Neral, and Citral	PC	Colorectal cancer (HCT116 and HT29)	Increased mitochondrial-mediated apoptosis, inhibited cell growth (IC_50_ 52.63 μM, and 91.5 μM for 72 h)	MTT	(Sheikh et al., 2017)
Tropolone monoterpene	Hinokitiol	Purity ≥ 90%	Human adenocarcinoma (A549)	Reduced cell migration and chemoprevention	MTT	(Jayakumar et al., 2018)
Bicyclic Monoterpene	Myrtenal	PC	Melanoma (B16F0, B16F10 and SkMel-5)	Decreased tumor cells migration and invasion	MTT	(Martins et al., 2019)
Oxygenated monoterpen	Carvone	PC	Breast ductal carcinoma (MCF-7)	Protective effect against tumor (IC_50_ 14.22 μM)	MTT	(Abbas, Kandil, & Abbas, 2020)

Note: PC = Pure Compound.

Similarly, Jayakumar et al. (2018) evaluated the effect of hinokitiol on the migration of A549 lung cancer cells. They observed that hinokitiol inhibited the migration of A549 cells via several mechanisms, including suppression of MMPs, induction of antioxidant enzymes (catalase and superoxide dismutase), and activation of caspases-9 and -3. It also significantly induced the cytochrome *c* expression (Jayakumar et al., 2018).

α-Thujone is another compound that has anticancer activity. In a study carried out by Pudełek et al. (2019), the effect of α-thujone on the malignancy of glioblastoma multiforme (GBM) cells was investigated. They found that α-thujone exerts the attenuating effect on the proliferation and viability of GBM cells. They also indicated that α-thujone exhibits anti-invasive and pro-apoptotic effects on GBM cells (Pudełek et al., 2019).

Furthermore, it has been reported by Potočnjak et al. (2018) that carvacrol exhibits anticancer activity against HeLa cells, a human cervical cancer cell line. Carvacrol induced cytotoxicity on HeLa cells and enhanced cisplatin (CP)-induced expression of light chain 3 beta in a manner dependent on the inhibition of mitogen-activated protein kinase (MEK). This compound, interestingly, also increased HeLa cells resistance on CP by suppressing apoptosis in an extracellular signal-regulated kinase (ERK1)/ERK2-independent manner and promoting the modulation of autophagy in an ERK1/ERK2-dependent manner (Potočnjak et al., 2018). In addition, carvacrol is a potential source of anticancer agent against the human gastric adenocarcinoma (AGS) cells. Carvacrol inhibited AGS cell proliferation and demonstrated genotoxic, ROS generating, and glutathione-reducing effects (Günes-Bayir et al., 2017).

Han et al. (2016) studied the ability of linalool-incorporated nanoparticles (LIN-NPs) as novel anticancer agents. They reported that LIN-NP had significant apoptotic and cytotoxicity activity against epithelial ovarian cancer cells (Han, Cho et al., 2016). Moreover, linalool has also been reported to exert anticancer activity against hepatocellular carcinoma HepG2 cells through modulation of Ras/MAPK and Akt/mTOR pathways (Rodenak-Kladniew et al., 2018).

In a study by Ma et al. (2016), the anticancer activity of perillyl alcohol was demonstrated. They found that perillyl alcohol has an inhibitory effect on protein synthesis of hypoxia-inducible factor 1α (HIF-1α) which causes a decrease in HCT116 cell growth in a xenograft colon tumor model. In addition, perillyl alcohol was also able to increase the expression of p53 and p21, decrease the expression of cyclin D1, c-Myc, and S-phase kinase-associated protein 2 (Skp2) and induce cell cycle catch in phase G1 in cancer cells (Ma et al., 2016).

The monoterpene myrtenal has also been reported to exhibit anticancer properties. Myrtenal plays an important role in controlling tumor malignancy and metastasis through inhibition of V-ATPase enzymes, disruption of H + flow to V-ATPase-dependent extracellular matrix, induction of autophagy, and alteration of ion gradients (Martins, Arruda, Costa, Jerdy, De Souza, Santos, De Freitas, Kanashiro, De Carvalho, Sant'anna, Antunes, Martinez-Zaguilan, Souad, Okorokova-Façanha, & Façanha, 2019). Lastly, bakuchiol, an example of meroterpene, has been reported to exert anticancer activity in the human stomach cancer cells. Preliminary investigation demonstrated that bakuchiol yield its anticancer activity via the induction of mitochondrial-dependent cell death and modulation of the phosphoinositide 3-kinase (PI3K)/protein kinase B (Akt) and mitogen-activated protein kinase (MAPK) pathways of Nagoya University-Gastric Cancer-3 (NUGC3) cells (Lv & Liu, 2017).

### Antimicrobial

The antimicrobial activity of EOs has been reported in many studies, but most of these studies attribute such action to the most common compounds without analyzing it independently (Guimarães et al., 2019). Infections caused by sensitive and drug-resistant bacteria or other microorganisms seriously harm human health and global public health. Therefore, finding and developing new antimicrobial agents to combat multidrug-resistant organisms is essential (Liu et al., 2020).

The use of terpene and terpenoid compounds as antimicrobial agents is very promising. It has been used in several industrial fields, including cosmetics, foods, and some health care products, such as hair tonics, toothpaste, eyelid cleansers, hair restorers, skin soaps, and body lotions (El Hachlafi et al., 2021). Terpene and terpenoid compounds have been reported to yield bactericidal effects (Guimarães et al., 2019). For example, limonene have synergistic modulation effects with gentamicin antibiotics in the inhibition of Gram-positive *Staphylococcus aureus* and Gram-negative *Escherichia coli* as and several resistant bacteria (Costa et al., 2019). In addition, a combination of limonene and ε-Polylysine demonstrates an additive and useful synergistic effect against *E. coli*, *S. aureus*, *Bacillus subtilis* and *Saccharomyces cerevisiae* (Zahi, El Hattab, Liang, & Yuan, 2017),

Monoterpene terpineol’s isomers (α-terpineol, terpinen-4-ol, and δ-terpineol) have a good inhibitory effect on several Gram-negative bacteria, especially *Shigella flexneri* with bacterial membrane permeability mechanisms resulting in the release of nucleic acids and proteins along with a decrease in membrane potential. In addition to that, the release of AKP indicates that the bacterial cell wall is destroyed, thus the potential use of terpineol in food as a natural antibacterial agent that is capable to destroy cell membranes and walls, resulting in bacterial cell death (Huang et al., 2021). Borneol and citral also have synergistic effects as bacteriostatic and antibiofilm agents in the packaging materials and common surface disinfectants. The synergistic effects of borneol and citral demonstrates a good elimination effect on the formation of biofilms produced by *Listeria monocytogenes* and *Pseudomonas aeruginosa*, most likely attributed to the ability of those two components to increase porosity and lytic effect on bacterial cell membranes as particularly suitable for use as promising food additives (Wang et al., 2021).

Hinokitiol is effective against Candida strain panels with several azole-resistant mechanisms and inhibits the growth of *Candida albicans* (Jin et al., 2021). The mechanism of action of hinokitiol is attributed to the chelated effect of intracellular iron fungus and inhibits the respiration of fungal cells but has little effect on the mammalian cells. It has been reported that hinokitiol also inhibits the activity of mitochondrial respiratory chains complex I and II and reduces the potential of mitochondrial membranes, thereby reducing intracellular ATP synthesis and increasing the intracellular reproductive stress. Thus, hinokitiol shows low potential to induce resistance in some Candida species and greatly improves the survival of Candida-infected *Galleria mellonella* (Jin et al., 2021). Furthermore, hinokitiol has also been demonstrated to yield prospective antibacterial effect with low or no negative effect on the human host or environment (Suzuki et al., 2019).

Other examples of EO with antimicrobial effect are eugenol (Devi, Nisha, Sakthivel, & Pandian, 2010). Eugenol showed rapid bactericidal action against *Salmonella enterica* serovar *Typhimurium*. Eugenol also demonstrated excellent bactericidal activity against strains of S. *aureus*. The compounds carveol, citronellol, and geraniol have a rapid bactericidal effect against *E. coli*. Furthermore, carvacrol, l-carveol, eugenol, *trans*-geraniol, and thymol, showed higher activity when compared to sulfanilamide. The inhibition of microbial growth and the death of bacterial cells is based on the loss of integrity in the function of cellular membranes (Guimarães et al., 2019). Terpenes and other terpenoid compounds, such as bakuchiol, α-pinene, linalool, champene, geraniol, 1,8-cineole, α-phellandrene, 3-carene, *p*-cymene, perillyl alcohol, bornyl acetate, and citral isomers, also have been reported to yield inhibitory effects on the growth of microorganisms (Table 2). These convincing data further indicate the potential use of these compounds as efficient antimicrobial agents in the food industry (Zahi et al., 2017), thus has a valuable contribution in supporting human health.

**Table 2:** Examples of terpene compounds in essential oils with the potential antimicrobial effects.

Classes	Compounds	Plant	Microorganism strain	Antimicrobial effects	Type of assay	Reference
Monoterpene hydrocarbons	Myrcene	PC	*Enterococcus faecalis* (ATCC 29212), *Streptococcus mutans* (ATCC 25175), *S. mutans* (ATCC 35668), *S. mutans* (ATCC UA159), and *Lactobacillus rhamnosus* (ATCC 53103)	Bacterial growth (MIC > 8 × 10^2^ CFU/ml)	Agar dilution	(Chaves-Quirós et al., 2020)
Terpineols Monoterpene	α-Terpineol	PC	*Escherichia coli* (CICC 21530), *Shigella flexneri* (CICC 21534), and *Salmonella enterica* (CICC 21513)	Good inhibitory effects against several gram-negative bacteria (MIC 1.531, 0.766, and 1.531 mg/ml)	Two-fold dilutions	(Huang et al., 2021)
Tropolone monoterpene	Hinokitiol	PC	*Bacillus subtilis*, *S. aureus*, *E. coli*, and *Pseudomonas aeruginosa*	(MIC 80, 160, 80, and 320 mg/ml)	Agar dilution	(Suzuki et al., 2019)
Bicyclic monoterpene	Borneol	Purity ≥ 98%	*E. coli* (ATCC 43894), *Listeria monocytogenes* (ATCC 19118), *S. aureus* (ATCC 23235), *P. aeruginosa* (ATCC 27853)	Bacteriostatic activity (MIC 1.25, 5, 5, 10 mg/ml)	Double microdilution	(Wang et al., 2021)
Terpenophenol	Bakuchiol	PC	*S. aureus* (ATCC29213), MRSA N315, MRSA NCTC10442, *E. coli* (ATCC25922), *P. aeruginosa* (ATCC9027), *Acinetobacter baumannii* (ATCC17978), *A. baumannii* (R2889), *Klebsiella pneumoniae* (ATCC10031), *K. pneumoniae* (ATCC14581)	Antibacterial activity (MIC 6.25, 3.125, 3.125, > 100, >50, >50, >50, 12.5, and > 50 μl/ ml)	Broth dilution method	(Li et al., 2021)
Cyclic monoterpene	d-Limonene	PC	*E. coli* (ATCC 8739), *S. aureus* (ATCC 6538), *B. subtilis* (ATCC 6633), *Saccharomyces cerevisiae* (ATCC 9763)	Exhibit strong synergistic (MIC 1, 1, 1, and 0.5 µg/ml)	Serial Dilution	(Zahi et al., 2017)
Acyclic monoterpene Alcohol	Geraniol	PC	*B. cereus* (MTCC 430), *E. coli*(MTCC 443)	Antimicrobial effect (MIC 1200 µl/ml all tested)	BHI broth	(Syed & Sarkar, 2018)
Oxygenated monoterpene	1,8-cineole	Purity 99%	*S. aureus* (ATCC 25923), MRSA clinical isolate, *P. aeruginosa* (ATCC 27853), *E. coli* (ATCC 25922), *K. pneumoniae* (ATCC 700603), *Enterococcus faecalis* (ATCC 51299) *Candida albicans* (ATCC 90028)	Effect against microorganisms (MIC 128, 128, 256, 32, 64, 128, and 32 g/l)	Serial double dilutions	(Şimşek & Duman, 2017)
Cyclic monoterpene	α-Phellandrene	Purity > 99%	*Penicillium cyclopium*	Inhibit the mycelia growth (MIC 1.7 ml/L)	Agar dilution	(Zhang, Sun, Chen, Zeng, & Wang, 2017)
Bicyclic Monoterpene	(+)-3-carene	Purity ≥ 90.0%	*Brochothrix thermosphacta* (ACCC 03,870), and *Pseudomonas fluorescens* (ATCC 13,525)	Had strong antibacterial activity (MIC 20 ml/L)	Agar dilution	(Shu et al., 2020)
Alkylbenzene Monoterpene	*p*-Cymene	*Salvia officinalis* (Essential oil)	*E. coli* (ATCC 8739), *S. aureus* (ATCC 6538), *C. albicans* (ATCC 10231)	Antibacterial activity (MIC 7.5, >15, and 3.75 μl/ml)	Broth microdilution	(Cutillas, Carrasco, Martinez-Gutierrez, Tomas, & Tudela, 2017)
Oxygenated monoterpene	Bornyl acetate	*S. officinalis* (Essential oil)	*E. coli* (ATCC 8739), *S. aureus* (ATCC 6538), *C. albicans*(ATCC 10231)	Antibacterial activity (MIC > 15, 15, and > 15 μl/ml)	Broth microdilution	(Cutillas et al., 2017)
Monoterpenoid phenol	Carvacrol	PC	*B. cereus* (MTCC 430), *E. coli* (MTCC 443)	Antimicrobial effect (MIC 400 µl/ml all tested)	BHI broth	(Syed & Sarkar, 2018)
Acyclic monoterpene Alcohol	Linalool	PC	*P. aeruginosa* (ATCC9027)	Antibacterial activity (MIC 431 µg/ml)	Medium dilution	(Liu et al., 2020)
Citral isomer	Citral	PC	*C. albicans* (SC5314), *C. tropicalis* (ATCC1369), *S. aureus* (ATCC25923)	Antimicrobial action (MIC 0.0313, 0.0156, and 0.0313 v/v%)	Serial microdilutions	(Gao et al., 2020)

Note: PC = Pure Compound.

### Anti-inflammatory

Recent decades have shown that terpenes and terpenoids are physiologically important to alleviate various symptoms caused by inflammation, most likely by inhibiting multiple pathological steps in the inflammatory process (Kim, Song, Cho, & Lee, 2020). Inflammation is a protective response of the host to non-self objects that are usually generated by microbial infection and/or tissue damage. Dysregulation of inflammatory responses can lead to acute and chronic inflammatory diseases that cause excessive or long-lasting tissue damage (Chen et al., 2017).

Macrophages, one of key immune cells, play a central role in many different immune pathological phenomena during inflammation, including overproduction of pro-inflammatory cytokines and inflammatory mediators, such as interleukin-1β (IL-1β), IL-6, tumor necrosis factor-alpha (TNF-α), and nitric oxide (NO) synthesized by non-reduced NO synthase (iNOS), and prostaglandin E2 (PGE-2) synthesized by cyclooxygenase-2 (COX-2). Lastly, a central transcription factor, nuclear-κB (NF-κB) factor, holds a central role in the expression of pro-inflammatory genes during inflammation (Liu, Zhang, Joo, & Sun, 2017).

Some cellular processes, including oxidative stress and autophagy, also play an essential role in inflammation. Reactive oxygen species (ROS), which results from several sources, including mitochondria, mediate increased leukocyte migration and junctional permeability through various signaling mechanisms. In addition, a recent study showed that ROS regulates the release of IL-1β by directly interfering with NF-κB signals (Warnatsch et al., 2017).

The application of terpenes and terpenoids to alleviate inflammation has been shown successful in the mitigation of respiratory inflammation, atopic dermatitis, arthritis, and neuroinflammation (Kim, Song et al., 2020). Table 3 shows that terpene compounds and terpenoids are beneficial as an anti-inflammatory in some disease conditions.

**Table 3:** Examples of terpene compounds in EO with the potential anti-inflammatory effects.

**Classes**	**Compounds**	**Plant**	**Type of inflammation**	**Anti-inflammatory effects**	**Assay/method**	**Reference**
Monoterpene hydrocarbons	Myrcene	PC	Renal tissues of rats	Inhibited the activities of inflammatory cytokine, pro-inflammatory signalling	ELISA	(Yang & Liao, 2021)
Cyclic monoterpene	R-(+)-limonene	Purity > 99%(Sigma Aldrich, St. Louis, MO, USA)	Gastric ulcer in rats	Decreased the levels of TNF-a, IL-6, and IL-1β and increased the level of IL-10	ELISA kits	(De Souza et al., 2019)
Bicyclic monoterpene	Borneol	PC	Acute pancreatitis mice model	Inhibited TNF-α, IL-1β, IL-6, and modulated Nrf2/NF-κB pathway	ELISA	(Bansod et al., 2021)
Tropolone monoterpene	Hinokitiol	PC	Inflammation in primary human keratinocytes	Inhibited LPS-mediated up-regulation of pro-inflammatory factors including tumor necrosis factor alpha, IL-6, and prostaglandin E2 (PGE2), and Sirt1 activity	Quantitative Real-Time PCR	(Lee et al., 2017)
Terpenophenol	Bakuchiol	*Psoralea corylifolia* (Seeds essential oil)	Cerebral ischemic injury in mouse BV-2 microglia	Exhibit its anti-inflammatory property via activating Nrf2 signaling	Immuno-fluorescence staining	(Xu, Gao, Wang, Yang, & Xie, 2021)
Bicyclic monoterpene	*α*-Pinene	PC	Focal cerebral ischemia–reperfusion in rats	Neuroprotective effect during ischemic stroke through attenuating neuroinflammation.	ELISA	(Khoshnazar, Parvardeh, & Bigdeli, 2020)
Acyclic monoterpene Alcohol	Linalool	*Boswellia carterii*	Ear edema model and a formalin-inflamed hind paw model	More potent pharmacological effects on hind paw inflammation and COX-2 overexpression	Immuno-histochemistry	(Li, Yang, Li, Zhang, & Tang, 2016)
Acyclic monoterpene Alcohol	Geraniol	PC	Lungs in mice model	Improved the inflammatory changes	ELISA	(Lin et al., 2021)
Cyclic monoterpene	α-Phellandrene	PC	Wound healing	Suppressed the overproduction of pro-inflammatory cytokines of IL-6 and TNF-α.	ELISA	(De Christo Scherer et al., 2019)
Hydroxylated monoterpene	Perillyl alcohol	Purity 96%	Lung tissue in rats	Inhibited cellular inflammation	ELISA	(Beik et al., 2021)
Citral isomer	Citral	Purity ≥ 95%	Hyperalgesia and pleurisy in mice	Anti-inflammatory activities	ELISA	(Campos, Lima, Trindade, Souza, Mota, Heimfarth, & Thangaraj, 2019)
Monoterpenoid phenol	Carvacrol	PC	Asthma in rats	Reduced of AEC, IgE, IL-4, IL-5, IL-13, TNF-α, IFN-γ, iNOS and MDA	Colorimetric and Quantitative Real-Time PCR	(Ezz-Eldin, Aboseif, & Khalaf, 2020)
Oxygenated monoterpene	1,8-cineole	PC	Upper Ileum Tissues	Prevented low-grade inflammation	Quantitative Real-Time PCR	(Jiang et al., 2021)

Note: PC = Pure Compound.

The prospective anti-inflammatory effect of myrcene in the treatment of renal inflammation was tested in the adrenalectomized rat models (Islam et al., 2020). The mechanisms were suggested to be associated with the downregulation of pro-inflammatory cytokine (IL-1β, IL-6, and TNF-α) and anti-inflammatory markers (IL-4 and IL-10) as well as the upregulation of endogenous antioxidants such as catalase (CAT), superoxide dismutases (SODs) and glutathione (GSH) (Islam et al., 2020).

Limonene, a cyclic monoterpene, has been reported to exert gastroprotective effect in rats (De Souza et al., 2019). The precise mechanism of its gastroprotective action remains unclear. However, current experimental evidence suggested that this effect may be due to its ability in the modulation of the oxidative stress (via upregulation of endogenous antioxidant glutathione peroxidase) and the alleviation of inflammatory responses (via inhibition of NF-κB-mediated gene expression) (De Souza et al., 2019).

Several terpenes and terpenoids such as (+)-α-terpineol, (−)-β-pinene, and (+)-α-pinene reported to reduce the expression of genes associated with inflammation (IL-4 and IL-13) and secretion of β-hexosaminidase in RBL-2H3 cells stimulated by LPS. The application of these compounds in the treatment of inflammatory conditions and the basis for the development of new anti-inflammatory drugs (Yang, Choi, Kim, Eom, & Park, 2021). Borneol, for example, has been reported to significantly increase the activation of nuclear factors E2-related factor 2 (Nrf2) and the expression of superoxide dismutase (SOD) 1 but at the same time downregulate the expression of NF-κB and p65. Treatment with borneol significantly inhibited the pro-inflammatory expression of cytokines and has the potential to alleviate cerulein-induced acute pancreatitis by reducing oxidative damage and inflammation of the pancreas in a manner dependent on the modulating of the Nrf2/NF-κB pathway (Bansod et al., 2021).

Another example is bakuchiol, a meroterpene in the class of terpenophenol, and hinokitiol, a natural monoterpenoid. Bakuchiol has been demonstrated to be effective in the suppression of pro-inflammatory cytokine expression and mitigation of delayed hypersensitivity responses compared to the control group (Kumar et al., 2021). Hinokitiol inhibits LPS-mediated regulation, including tumor necrosis factor-alpha, IL-6, and prostaglandin E2 (PGE2). Hinokitiol is suggested to act through the inhibition of LPS-mediated proinflammatory signals via the activation of histone sirt1 deacetylase in primary human keratinocytes. Hence, hinokitiol has been suggested as a potential therapeutic agent in the treatment of inflammatory skin diseases such as psoriasis (Lee, Moon, Lee, & Park, 2017).

### Antioxidant

Some EOs have an important role in reducing oxidative stress and often used to prevent several chronic diseases. Chamazulene, a bicyclic sesquiterpene derivative from EOs of *Matricaria chamomilla,* was able to balance ROS level on bovine aortic endothelial cells-1 (BAECs), which increase due to high glucose and H*_2_*O_2_ treatment (Querio et al., 2018). Ursolic acid which isolated from *Entada abyssinica* is a pentacyclic triterpenoid carboxylic acid. Ursolic acid has antioxidant activity with IC_50_ 1.43 ± 0.080, 2.87 ± 1.19, and 7.04 ± 1.29 µg/ml by using FRAP, DPPH, and ABTS method, respectively (Dzoyem et al., 2017). FRAP, DPPH, and ABTS are radical compound which can assist in the measurement of antioxidant activity through color change. DPPH will change into a non-radical compound (diphenylpicrylhydrazine), characterized by a change in the color of the solution from purple to pale yellow, if its free electrons bind to the hydrogen atom of an antioxidant compound. In the ABTS method, the solution will change from blue or green to colorless due to the acceptance of proton donors from antioxidant compounds. Meanwhile, in the FRAP method, the solution will change color from yellow to blue when ferri-tripyridyl-triazine (Fe(III)TPTZ) becomes ferro-tripyridyl-triazine (Fe(II)TPTZ) due to electron transfer from antioxidant compounds (Moon & Shibamoto, 2009).

Some terpeneoids (α-pinene, limonene, nerol, terpinol, geraniol, linalool, and myrcene) are responsible for specific aroma in wine. α-Pinene showed the strongest radical scavenging effect with IC_50_ 12.57 ± 0.18 mg/ml, followed by limonene and nerol with IC_50_ 13.35 ± 0.26 and 26.08 ± 2.28 mg/ml, respectively. This is in line with the result of reducing power assay, where α-pinene has the greatest reducing power among others (213.7 ± 5.27 mg/ml), followed by limonene (133.48 ± 6.22 mg/ml), nerol (79.15 ± 3.75 mg/ml), terpinol (73.92 ± 3.34 mg/ml), geraniol (73.78 ± 3.94 mg/ml), linalool (43.97 ± 1.09), and the lowest was myrcene (21.59 ± 1.14 mg/ml) (Wang et al., 2019).

*Magnolia biondii*, one of traditional Chinese herb, contain many active components in its essential oil. Of all, α-terpineol showed the highest % rate of scavenging (84.1%) followed by c-cadinene (83.9%), geraniol (75.5%), linalool (65.5%), and citronellol (58.1%) by DPPH assays. Similar results were also obtained in the ABTS assays, where these five components had the highest scavenging activity compared to the others, ranging from 89.1% to 83.0% (Nie et al., 2020). Essential oil of *Cinnamon* contains cinnamaldehyde which has high scavenging rate of 93.0% by using DPPH assay (Farag, Alagawany, & Tufarelli, 2017). Eugenol and isoeugenol which found in a variety of plants including cinnamon, basil, clove, spices, and nutmeg have quite promising antioxidant activity, with EC_50_ of 22.1 ± 3.5 and 17.6 ± 4.1 µg/ml for DPPH assay, 146.5 ± 5.6 and 87.9 ± 4.7 µg/ml for ABTS assay, and 11.2 ± 1.5 and 18.4 ± 1.2 µg/ml for FRAP assay, respectively. These results indicate that isoeugenol has slightly higher scavenging activity than eugenol (Zhang, Zhang, Xu, & Hu, 2017).

### Anti-allergic

Allergic diseases are characterized by inflammation with infiltration of T cells and granulocytes (eosinophils, neutrophils, and mast cells). Mast cells play a major role in almost all allergic diseases, generally in the end reaction of allergic disease (Modena, Dazy, & White, 2016). Mast cells begin to synthesize prostanoids and proinflammatory leukotrienes, and subsequently produce inflammatory cytokines, such as IL-4, IL-5, IL-13, IL-1α/β, thereby stimulating the activation of other cells such as neutrophils, monocytes, basophils, eosinophils and lymphocytes. Anti-allergic compounds derived from plants, animals, and microbes, with various mechanisms of action such as binding to the epitope present in allergens, affecting gut microbiota and intestinal epithelial cells, changing antigen presentation and T cell differentiation, and inhibiting effector cell degranulation (Yang, Li, Xue, Huang, & Wang, 2021).

Some terpenes and terpenoids have been studied for anti-allergic activity. Atractylone (Atr), one of sesquiterpene components from *Atractylodes japonica*, can inhibit rat peritoneal mast cells (RPMC) degranulation, tryptase and histamine release, and intracellular calcium levels. Also, reduced tryptase and histamine release from PMA plus A23187-stimulated HMC-1 cells, led to decreased actvity and expression of histidine decarboxylase in activated HMC-1 cells. Atr decreased levels of histamine, IgE, IL-4, IL-5, IL-6, vascular endothelial growth factor, and IL-13 in the serum of PCA-induced mice. Atr has activity as a treatment for allergic reactions mediated by mast cells (Han, Moon et al., 2016). Carvone (S- and R-) at concentrations 10 mg/kg via oral route has been reported to reduce the total eosinophil and leukocytes counts in the murine model of airway allergic inflammation induced by sensitization and challenge with ovalbumin (OVA). S-carvone significantly increased the concentrations of IFN- and neutrophil counts in the BAL of allergic mice. R-carvone could stimulate IL-10 synthesis and inhibit IgE secretion (Ribeiro-Filho et al., 2020). β-Amyrin, 2α,3α,23- trihydroxyursa-12,20(30)-dien-28-oic acid, and euscaphic acid at 10 µM, inhibited histamine release with percentage of inhibitions of 46.7, 57.9, and 54.2%, respectively. In addition, β-amyrin and 2α,3α,23- trihydroxyursa-12,20(30)-dien-28-oic acid showed strong inhibition of TNF- α and IL-6 in the test for pro-inflammatory cytokines (Choi, Kim, Kim, & Kim, 2016).

Citronellol, one of major component from geranium EOs, at concentrations 0.5 mM can inhibit degranulation of mast cells by 69.4% and significantly inhibit IgE- induced tumor necrosis factor -α (TNF-α) production (Kobayashi et al., 2016). Vernodalin isolated from *Vernonia amygdalina* increase filaggrin (FLG) mRNA expression levels and reduce IL-33mRNA expression in mice (vs. control group) also significantly reduce the dermatitis score (vs. control) in regard to ear skin lesions at 100 µg/ml via topical administration (Hirota & Ngatu, 2018). Mojabanchromanol, a constituent isolated from *Sargassum horneri*, has anti-allergy effect on bone marrow-derived cultured mast cells. Mojabanchromanol shows inhibitory effect on the β-hexosaminidase release and inhibited the mRNA expression levels of allergic cytokines in bone marrow cultured mast cells (BMCMCs) (Kim, Han et al., 2020).

## Bioaccessibility and bioavailability of terpenes and terpenoids

Bioavailability is a term that refers to a measure of the rate and fraction of the initial dose of a drug that successfully reaches the site of action or body fluids of the target drug to produce the desired biologic effect (Currie, 2018). Bioavailability includes two interrelated parts, namely: bioaccessibility and bioactivity. Bioaccessibility is defined as the quantity or fraction released from the food matrix in the digestive tract that is available for absorption (Thakur et al., 2020).

Studies on the bioavailability and bioaccessibility of a compound are generally carried out by in vitro or in vivo methods. Simulation of the condition of the gastrointestinal system by adjusting the pH, digestive enzymes and some chemical reactions that occur, is the most widely used in vitro digestion method (Jones, Caballero, & Davidov-Pardo, 2019). In vitro methods can also be carried out using a Caco-2 cell model wherein the absorbed target compound is collected on the basolateral side of the monolayer model cell (Jones et al., 2019). In vivo methods were carried out using animal models prior to clinical studies.

The matrix of medicinal plants is related to the bioavailability of the terpenes they contain because they must pass through digestion in the mouth and stomach before accessing the small intestine. Furthermore, these compounds will undergo mechanical and enzymatic actions, changes in pH conditions, as well as water-soluble transformations, which occur mainly in the liver and other tissues such as the lungs, gastrointestinal, kidney, blood and brain. Terpenes describe high lipophilic behavior that influences their solubility in the aqueous phase of the gut lumen, thus incorporation with the lipid phase becomes very important in the bioavailability of terpenes, either during digestion or during food processing (Mouhid et al., 2017). Even though intravenous administration is assumed has maximal (100%) bioavailability of EOs, skin application, oral intake, and inhalation are the primary intake routes of EOs (Stevanovic et al., 2020). Some studies have been conducted in order to understand bioavailability and bioaccessibilty of terpenes and terpenoids (Table S1).

Terpenoids easily enter the body by penetration through the skin due to their lipophilic properties although the amount depends on the area of skin applied, skin characteristics, concentration of compounds and exposure time. Following oral administration of terpenoids, the upper gastrointestinal tract did not have a significant role in the absorption. However, using the route of inhalation, terpenoids may be absorbed by the lungs so that they are available systemically. This absorption depends on the type of compound and the respiratory mechanics of the subject. Enterohepatic circulation, pulmonary and renal excretion play a role in the elimination of terpenoids in the form of feces, expired air, and urine. A minor part is eliminated along with the feces, and the rest through the urine as terpene conjugates and in the expired air with CO_2_. Drug biotransformation occurs in the liver by phase I and phase II reactions, but in many terpenoids it takes only one phase to eliminate these volatile compounds (Jäger and Höferl, 2020, Kohlert et al., 2000). Camphor undergoes phase II metabolism to 5-*exo*-hydroxycamphor. Menthol and peppermint oil are excreted through the kidneys as menthol glucuronide. Terpenoids have high clearance and a short elimination half-life making accumulation of their metabolites are impossible.

Some EOs were used as penetration enhancer of drug via transdermal administration. Galangal EO was added to flurbiprofen microemulsion gel to increase bioavailability of flurbiprofen as anti-inflammatory drugs (Dong et al., 2020). Menthol and menthone can enhance penetration of ligustrazine hydrochloride, an anti-platelet aggregation, on transdermal absorption (Wang et al., 2017). In the oral administration, the main absorption site, the intestine, the absorption rate of EOs depends on lipophilicity, polarity, solubility, and molecular weight (Stevanovic et al., 2020). Due to different physiological and chemical condition of absorption site in GI tract, different rates of absorption can be happened in different terpenes. For example, sesquiterpene lactones and diterpene lactones, which have similar structures, have often been reported to have changeable pharmacokinetics, particularly unstable absorption and extensive metabolism. Although this compound is quite permeable in the intestinal epithelium, its absorption can be unstable due to the influence of gastrointestinal pH and efflux transporters (P-glycoprotein). P-gp expression increases from the proximal to the distal end of the small intestine causing a different absorption area of the P-gp substrate. Sesquiterpene lactone and diterpene lactone tend to be absorbed most effectively in the duodenum, followed by the jejunum, ileum and colon (Liu et al., 2019). In addition, differences in pH in the GI tract also affect terpene absorption, where the pH of the stomach, duodenum and colon is 1.2, 5.5 and 7.0, respectively (Wang et al., 2020).

## Potential and limitation application of EO as natural food preservatives

One of the crucial matters in the food industry is to provide safe and healthy food. Thereby, to improve safety and extend the shelf life of food products, many synthetic preservatives have been permitted to be used in the food industry to prevent contamination of foodborne pathogens and/or to control spoilage (Yousefi, Khorshidian, & Hosseini, 2020). Nevertheless, following the consumers' preference to consume food products with natural substances, the food industry has also been exploiting the use of natural preservatives in their products. In this sense, EOs exhibit various activities including antibacterial, antioxidant and antifungal properties that are considered as alternative eco-friendly food preservatives (Pandey et al., 2017).

Several EOs and their constituents were approved by the United States Food and Drug Administration (FDA) as Generally Recognized as Safe (GRAS) status to be used as flavorings and food preservatives (Ben Jemaa et al., 2017). The registered EOs have GRAS status including basil, cinnamon, clove, coriander, ginger, lavandin, menthol, nutmeg, oregano, rose, sage, and thyme EOs. Likewise, the reported EO constituent comprise carvacrol, carvone, citral, cinnamaldehyde, eugenol, limonene, linalool, thymol, and vanillin. The main constituents of EOs such as terpenes, play an important role in food safety without affecting the quality (Falleh et al., 2020).

EOs have the potential to be used as a food preservative for various food products (Table 4) such as meat, breads, grains, fruits, vegetables, milk, and dairy products (Pandey et al., 2017). Various pathogens including *E. coli*, *Clostridium* spp., *Salmonella* spp., *Campylobacter jejuni*, *Aeromonas hydrophila*, *S. cerevisiae*, *Penicillium expansum*, and *Listeria monocytogenes* involved in the spoilage of food products. Among them, *L. monocytogenes* has been reported as the main causative agent of serious diseases in humans and animals (Yousefi et al., 2020).

**Table 4:** Examples of conducted studies on preservation of food products by essential oils.

Food product	Investigated essential oil	Main bioactive compounds	Key findings	Reference
Bread	*Cymbopogon citratus* (lemongrass)	(z)-Citral (62.58%), *cis*-verbenol (6.29%), geranyl acetate (5.36%), isoeugenol (4.52%), caryophyllene (3.91%)	The vapor of lemongrass EO (750 µl of EO/L_air_) could inhibit *Penicillium expansum* inoculated on bread for 21 days at 20 °C.	(Mani López, Valle Vargas, Palou, & López Malo, 2018)
Cake	*Thymus vulgaris*	Thymol (53.57%), *p*-cymene (15.51%), limonene (7.14%), carvacrol (6.93%), *trans*-caryophyllene (3.26%), α-pinene (2.80%)	Addition of encapsulated thyme essential oil (0.60 mg/ml) in the cake formulation enhanced the shelf life of the product for 30 days of storage.	(Gonçalves et al., 2017)
Dry fruits	*Mentha cardiaca* L.	Carvone (59.6%), limonene (23.3%), β-myrcene (2.5%), 1,8-cineole (2.1%), β-bourbonene (1.5%), *cis*-dihydrocarvone (1.5%)	*M. cardiaca* EO showed strong antifungal activity (MIC 1.25 µl/ml) against biodeterioration fungi of dry fruits and potentially to reduce aflatoxin secretion.	(Dwivedy, Prakash, Chanotiya, Bisht, & Dubey, 2017)
Green gram seeds	*Lippia alba*	Geranial (36.94%), neral (29.32%), myrcene (18.65%), α-caryophyllene (2.07%), eugenol (1.82%), α-phelandrine (1.02%)	Utilization of a dose of 80 μl/0.25 L of *Lippia alba* oil in green gram seeds significantly inhibited the proliferation of fungal and production of aflatoxin B_1_ without affecting the seed germination rate during storage.	(Pandey, Sonker, & Singh, 2016)
Orangina fruit juice	*Eucalyptus globulus* essential oil	1,8-cineole (94.03%), α-pinene (2.93%), γ-terpinene (1.93%), α-phellandrene (0.59%), β-pinene (0.20%), myrcene (0.19%)	EGEO (0.8 to 4 μl/ml) was effective and potent to reduce *S. cerevisiae* growth in the fruit juice of Orangina.	(Boukhatem et al., 2020)
Pineapple juice	*Cymbopogon citratus* D.C. Stapf. essential oil (CCEO)	Geraniol (46.16%), neral (31.74%), geranyl-acetate (4.34%), caryophyllene (2.02%), 6-methyl-5-hepten-2-ona (1.77%), dipentyl-ketone (1.06%), linalool (1.03%)	The incorporation of CCEO in pineapple juice at all tested concentrations (5, 2.5, and 1.25 μl/ml) caused a decrease in viable counts of *E. coli, L. monocytogenes,* and *Salmonella enterica*.	(Leite et al., 2016)
Chicken breast fillets	*Zingiber officinale*(Ginger)	α-Zingiberene (24.96%), β-sesquiphellandrene (12.74%), sesquisabinene hydrate (6.19%), camphene (5.90%), zingiberenol (4.26%), (E)-citral (3.93%), sabinene (3.75%), (E)-farnesene (3.73%), and italicene (3.21%)	Ginger essential oils nanoemulsion (6%) significantly reduced *L. monocytogenes* growth in refrigerated chicken filets during 12 days of storage.	(Noori, Zeynali, & Almasi, 2018)
Chicken meatballs	*Ziziphora clinopodioides*	Carvacrol (65.22%), thymol (19.51%), *p*-cymene (4.86%),and γ-terpinene (4.63%)	*Z. clinopodioides* EO (0.3%) efficiently inhibited the growth of *L. monocytogenes* in chicken meatball during 12 days storage 4 °C without any unfavorable sensory properties.	(Shahbazi, Karami, & Shavisi, 2018)
Dry Fermented Sausages	*Juniperus communis* L.	β-myrcene (14.12%), sabinene (9.51%), d,l-limonene (8.36%), 4-terpineol (6.88%), α-amorphene (5.43%), β-pinene (5.39%), caryophyllene (3.94%), *p*-cymene (3.92%), germacrene D (3.81%),	*Juniperus communis* EO can be utilized to control foodborne pathogens (*L. monocytogenes, Salmonella* spp*., E. coli,* and sulfite-reducing clostridia) in dry fermented sausages during storage period (225 days). The sample with 0.10 µl/g of *Juniperus communis* EO had untypical flavor.	(Tomović et al., 2020)
Ground beef	*Melaleuca alternifolia* (tea tree)	Terpinen-4-ol (43.1%), γ-terpinene (22.8%), α-terpinene (9.3%), α-terpineol (5.2%), terpinolene (3.5%), and α-pinene (3.0%)	The incorporation of 1.5% v/w *Melaleuca alternifolia* EO in ground beef was effective against *L. monocytogenes* with MIC and MBC values of 0.10 μl/g and 0.15 μl/ml, respectively. *Melaleuca alternifolia* EO was not significantly effective in the sample with the suspension at 1.5 × 10^8^ CFU/ml.	(Silva, Figueiredo, Stamford, & Silva, 2019)
Minced beef meat	*Citrus limon* (lemon)	β-Pinene (25.44%), limonene (39.74%), linalool (2.16%), α-terpineol (7.30%), linalyl acetate (3.01%), acetate geranyl (3.03%), nerolidol (6.91%), acetate neryl (1.74%), and farnesol (4.28%).	The application of *Citrus limon* EO (0.06 and 0.312 mg/g) can be considered as a natural substance in controlling growth of *L. monocytogenes* during storage at 4 °C of minced beef meat.	(Ben Hsouna, Ben Halima, Smaoui, & Hamdi, 2017)
Sausages	Thyme essential oil	Thymol (38.2%), *p*-cymene (25.4%) and terpineol with γ-terpinene (16.2%), α-pinene (2.2%)	Thyme EO inhibited development of *L. monocytogenes* in sausages. The main constituents of thyme EO related to thymol that disrupts membrane cells and reduces the activity of ATPase of *L. monocytogenes.*	(Blanco-Lizarazo, Betancourt-Cortés, Lombana, Carrillo-Castro, & Sotelo-Díaz, 2017)
Turkey meat	*Zataria multiflora* Boiss and *Bunium persicum* Boiss	*Zataria multiflora* Boiss: carvacrol (51.55%), thymol (25.49%), *p*-cymene (5.23%), and γ-terpinene (4.44%). *Bunium persicum* Boiss: cumic aldehyde (38.39%), *p*-cymene (18.36%), and 2-caren-10-al (13.26%)	Nanoemulsion of BEO and ZEO could extend the shelf life of turkey meat to 9 days. The chitosan-loaded nanoemulsion containing ZEO 1% provided the best antimicrobial activity. Nanoemulsion containing BEO and ZEO significantly decreased the population of *Salmonella* Enteritidis and *L. monocytogenes* about 3 log CFU/g and 2 log CFU/g, respectively.	(Keykhosravy, Khanzadi, Hashemi, & Azizzadeh, 2020)
UHT milk	*Syzygium aromaticum*(clove)*, Cinnamomum zeylanicum* (cinnamon)*, Myrtus communis* (myrtle)*,* and *Lavandula stoechas* (lavender)	*Syzygium aromaticum* (clove): eugenol (74.5%), caryophyllene (20.4%), aceteugenol (2.6%), β‐selinene (2%). *Cinnamomum zeylanicum* (cinnamon): Cinnamaldehyde (89%), camphene (3.5%), α‐terpineol (1.6%), 1,8‐cineole (1.6%). *Myrtus communis* (myrtle): Butanoic acid, 2-methyl, 2-methylbutyl ester (74.6%), 1,8‐cineole (11.5%), d‐limonene (6.5%), linalool (1.9%). *Lavandula stoechas* (lavender): camphor (35.4%), α‐fenchone (32.5%), 1,8‐cineole (7.4%), camphene (4.3%), α‐pinene (2.8%).	The combination of 2.4% *S. aromaticum*, 38.2% *L. stoechas*, and 59.4% *C. zeylanicum* significantly restricted the viability of E. coli to 1 × 10^6^ CFU.ml^−1^. These findings are to be taken into consideration for a successful application of these essential oils as food preservatives in milk and dairy industries.	(Falleh et al., 2019)

In a study by Khaleque et al. (2016), the effect of clove and cinnamon EO against *L. monocytogenes* in ground beef were determined (Khaleque et al., 2016). They found that 10% of crude and commercial clove EOs could be effective to decrease contamination and growth of *L. monocytogenes*. Furthermore, recent findings indicated that *Eucalyptus globulus* EO could reduce *S. cerevisiae* growth in the Orangina fruit juice (Boukhatem, Boumaiza, Nada, Rajabi, & Mousa, 2020).

Regardless, the main limitations for using EOs as food preservatives are their organoleptic effects (Falleh et al., 2020). In fact, EOs intense aroma sometimes adversely affects the organoleptic characteristics of food matrices. Several strategies have been considered to overcome this hurdle. Carpena et al. (2021) suggested that encapsulation is considered as a promising technique to minimize the EOs organoleptic impact. EOs may be used in active packaging as encapsulated molecules into microemulsions or nanoemulsions (Carpena et al., 2021). Active packaging is an innovative packaging technology with an extra function to extend product shelf life, ensure food quality and safety, and improve the appearance of the packaged food (Fang, Zhao, Warner, & Johnson, 2017). In addition, it also has been reported that *Thymus capitatus* (thyme EO) and its nanoemulsion were able to entirely inhibit Gram positive bacterial (*S. aureus, Bacillus licheniformis, and Enterococcus hirae*) in contaminated milk (Ben Jemaa et al., 2017).

## Concluding remarks and future perspectives

The information compiled in this review demonstrates that EOs and their main active constituent(s) are crucial in pharmaceutical and medical industries with several potential activities such as anticancer, antimicrobial, anti-inflammatory, antioxidant, and anti-allergic. Nevertheless, more studies are necessarily required to understand the mechanism behind the biological properties of EOs. Major bioactive compounds of EOs need to be clearly elucidated to improve their potential efficiencies in disease management. In addition to their function in health, EOs show high potential to be used as natural food preservatives in the food industry. However, organoleptic impact and probable contamination of EOs in food products have been suggested to limit their use. Indeed, careful and thorough investigations are urgently needed to reduce the disadvantages of EOs to meet the needs for food industry applications. Accordingly, scientific efforts need to be further initiated and developed to evaluate the effects of incorporating EOs into packaging systems and to ensure their safety for the consumers and the environment.

## Declaration of Competing Interest

The authors declare that they have no known competing financial interests or personal relationships that could have appeared to influence the work reported in this paper.
