# Supplementary material for: Terpenes and terpenoids as main bioactive compounds of essential oils, their roles in human health and potential application as natural food preservatives
Source: Food Chem X. 2022 Jan 19;13:100217. doi: 10.1016/j.fochx.2022.100217 (PMC9039924; doi:10.1016/j.fochx.2022.100217)
Supplement: Supplementary data 1 [file mmc1.docx]

**Supplementary aterial**

**Figure Legends**

**Fig. S1.** Biosynthesis pathways of terpenes and terpenoids.

**Fig. S2.** Scheme of general workflow followed in terpenes and terpenoids analysis on four important steps.

**Fig. S3.** The methods for extraction, separation, isolation, purification, and determination of the structural compounds of terpenes and terpenoids.


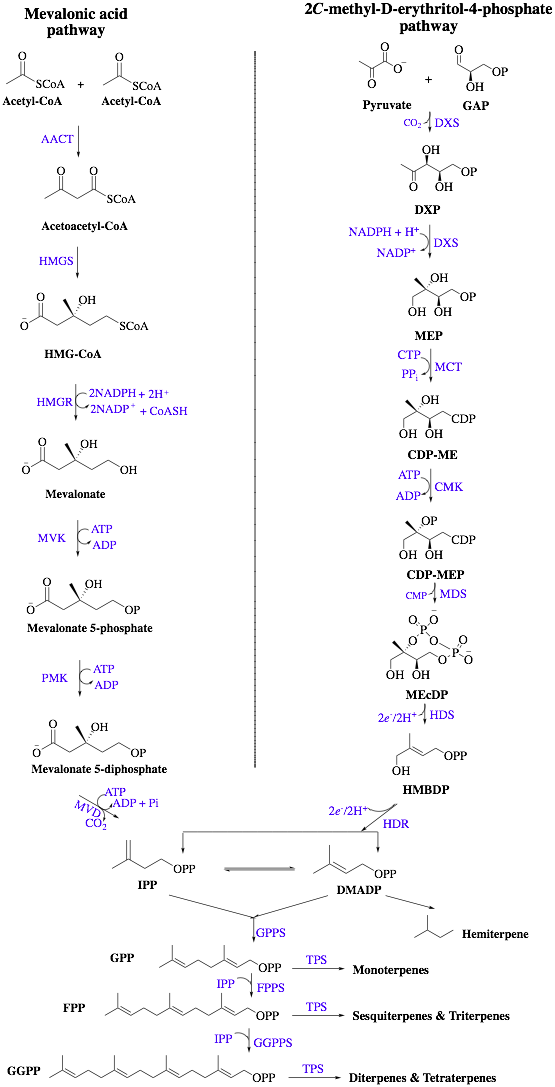


**Fig. S1.** Biosynthesis pathways of terpenes and terpenoids.


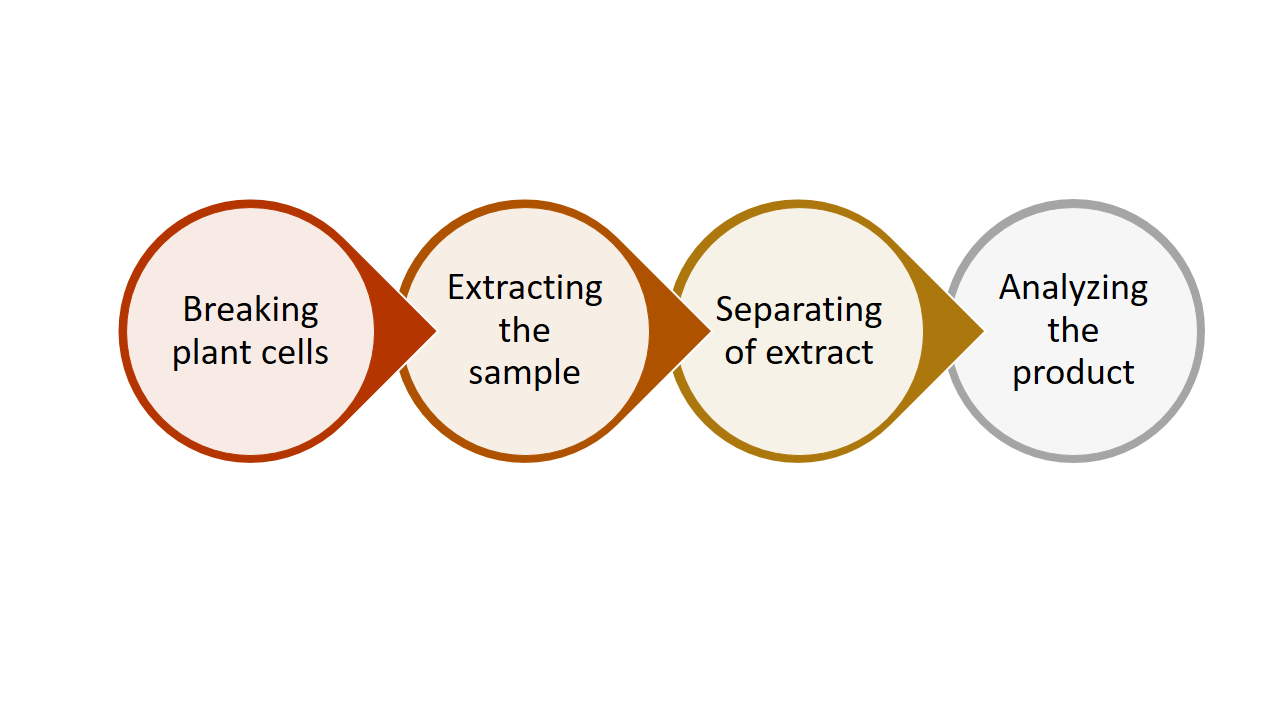


**Fig. S2.** Scheme of general workflow followed in terpenes and terpenoids analysis on four important steps.


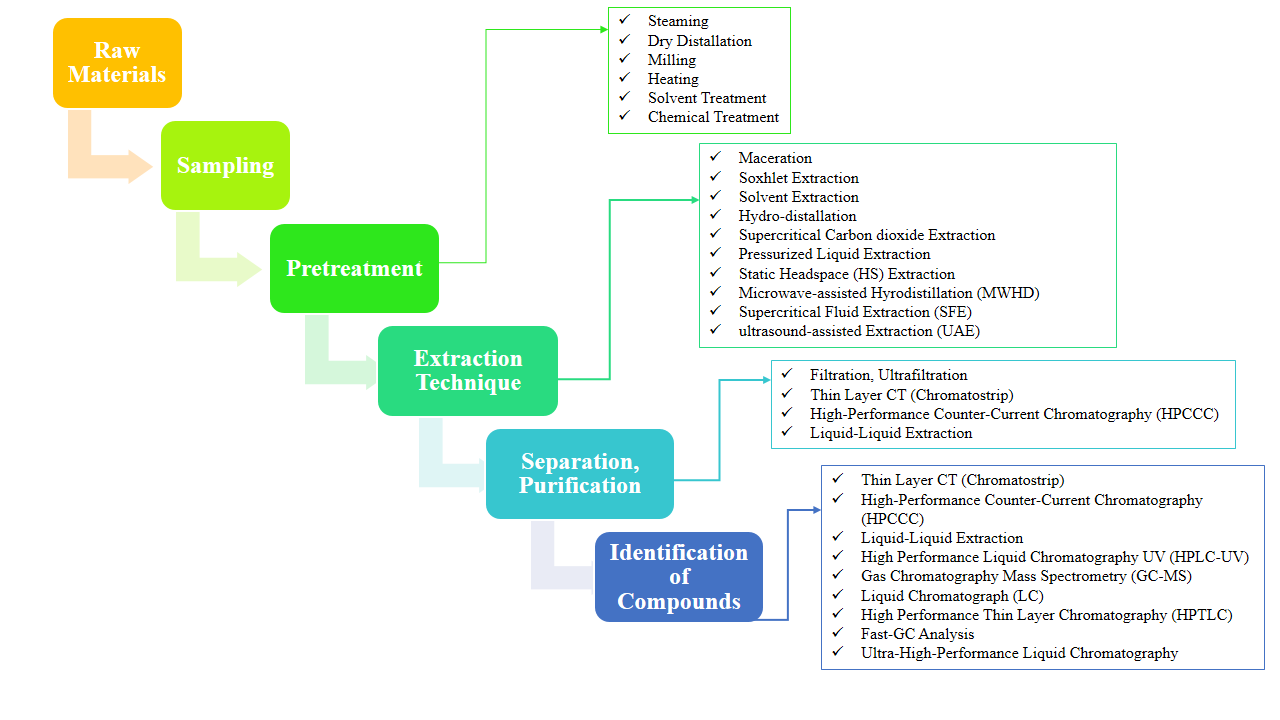


**Fig. S3.** The methods for extraction, separation, isolation, purification, and determination of the structural compounds of terpenes and terpenoids.

**Tabel S1.** Studies on bioavailability and bioaccessibility of some terpenes and terpenoids in EOs

| **Terpene/Terpenoids** | **Method** | **Results** | **References** |
| --- | --- | --- | --- |
| Mastiha powder (a natural resin rich in triterpenes, mastihadienonic, isomastihadienonic, oleanonic and moronic acid) | Bioavailability in human plasma (postprandial kinetic study) using UHPLC-HRMS/MS analysis | Major terpenes  are bioavailable since 0.5 h after administration, reaching a peak between 2-4 h. Serum resistance to oxidation starts to increase from 0.5 h and reaches statistical  significance at 4 h and remains  statistically significant until 24 h. | (Papada et al., 2018) |
| Rosemary extract (carnosic  acid, carnosol, rosmanol and its isomers (epiisorosmanol and epirosmanol), 12-methoxycarnosic acid, rosmadial, rosmaridiphenol, hinokione and miltipolone) | Absorption and bioavailability in Caco-2 cell monolayer model, both in a free form or liposomed. | The encapsulated form showed absorption values lower than the free extract. The permeability for epirosmanol, epiisorosmanol and rosmanol were decreased dramatically, while 12-methoxy carnosic acid, carnosic acid, rosmaridiphenol, miltipolone and hinokione showed no absorption in the encapsulated extract, as same as the triterpenoids. | (Pérez-Sánchez et al., 2017) |
| *Artichoke Extract* (The  phenolic acids and  sesquiterpene lactones  was detected, being 13.77  and 11.99 mg·g^−1^ after 20 h  of in vitro large  intestine fermentation. | The bioaccessibility and gut bioavailability of artichoke constituents were evaluated by combining in vitro digestion and large intestine fermentation, metabolomics, and Caco-2 human intestinal cells model. | The relatively high bioavailability values recorded for flavonols, phenolic acids, and sesquiterpene lactones (from 71.6% up to 82.4%) demonstrated that these compounds are able to be transported through the Caco-2 monolayer | (Rocchetti et al., 2020) |

**References**

Papada, E., Gioxari, A., Brieudes, V., Amerikanou, C., Halabalaki, M., Skaltsounis, A. L., Smyrnioudis, I. & Kaliora, A. C. 2018. Bioavailability of Terpenes and Postprandial Effect on Human Antioxidant Potential. An Open-Label Study in Healthy Subjects. *Molecular Nutrition & Food Research,* 62**,** 1700751.

Pérez-Sánchez, A., Borrás-Linares, I., Barrajón-Catalán, E., Arráez-Román, D., González-Álvarez, I., Ibáñez, E., Segura-Carretero, A., Bermejo, M. & Micol, V. 2017. Evaluation of the intestinal permeability of rosemary (Rosmarinus officinalis L.) extract polyphenols and terpenoids in Caco-2 cell monolayers. *PLOS ONE,* 12**,** e0172063.

Rocchetti, G., Giuberti, G., Lucchini, F. & Lucini, L. 2020. Polyphenols and Sesquiterpene Lactones from Artichoke Heads: Modulation of Starch Digestion, Gut Bioaccessibility, and Bioavailability following In Vitro Digestion and Large Intestine Fermentation. *Antioxidants,* 9**,** 306.
